# The Coordinated P53 and Estrogen Receptor Cis-Regulation at an FLT1 Promoter SNP Is Specific to Genotoxic Stress and Estrogenic Compound

**DOI:** 10.1371/journal.pone.0010236

**Published:** 2010-04-21

**Authors:** Yari Ciribilli, Virginia Andreotti, Daniel Menendez, Jan-Stephan Langen, Gilbert Schoenfelder, Michael A. Resnick, Alberto Inga

**Affiliations:** 1 Unit of Molecular Mutagenesis and DNA Repair, National Institute for Cancer Research, IST, Genoa, Italy; 2 Laboratory of Molecular Genetics, National Institute of Environmental Health Sciences (NIEHS), National Institutes of Health (NIH), Research Triangle Park, North Carolina, United States of America; 3 Institute of Clinical Pharmacology and Toxicology, Charité-Universitaetsmedizin Berlin, Berlin, Germany; 4 Institute of Pharmacology and Toxicology, Julius-Maximilians-Universität, Würzburg, Germany; 5 Centre for Integrative Biology, CIBIO, University of Trento, Trento, Italy; Institute of Genetics and Molecular and Cellular Biology, France

## Abstract

**Background:**

Recently, we established that a C>T single nucleotide polymorphism (SNP) in the promoter of the VEGF receptor FLT1 gene generates a ½ site p53 response element (RE-T) that results in p53 responsiveness of the promoter. The transcriptional control required an estrogen receptor (ER) ½ site response element (ERE1) 225 nt upstream to the RE-T.

**Methodology/Principal Findings:**

Here we report the identification of a second ER ½ site (ERE2) located 145 bp downstream of the RE-T and establish that both EREs can impact p53-mediated transactivation of FLT1-T in a manner that is cell type and ER level dependent. Gene reporter assays and ChIP experiments conducted in the breast cancer-derived MCF7 cells revealed that the ERE2 site was sufficient for p53-mediated ERα recruitment and transactivation of the FLT1-T promoter/reporter construct. Surprisingly, unlike the case for other p53 target promoters, p53-mediated transactivation of FLT1-T constructs or expression of the endogenous FLT1 gene, as well as binding of p53 and ER at the promoter constructs, was inducible by doxorubicin but not by 5-fluorouracil. Furthermore, ER activity at FLT1-T was differentially affected by ER ligands, compared to a control TFF1/pS2 ER target promoter. The p53-related transcription factors (TFs) p73 and p63 had no effect on FLT1 transactivation.

**Conclusions/Significance:**

We establish a new dimension to the p53 master regulatory network where p53-mediated transcription from a ½ site RE can be determined by ER binding at one or more *cis*-acting EREs in manner that is dependent on level of ER protein, the type of ER ligand and the specific p53-inducing agent.

## Introduction

The Vascular Endothelial Growth Factor Receptor-1 (VEGFR-1), commonly known as FLT1, is a high affinity VEGF receptor belonging to the VEGFR transmembrane receptor tyrosin kinase family, expressed in a variety of cell types, including endothelial cells, hematopoietic stem cells, leucocytes, and osteoblasts [1]. The FLT1 protein, which possesses higher affinity for VEGFA but weak tyrosine kinase activity, or none at all in the case of the soluble form (sFLT1), can act as inhibitory or decoy [2] to the FLK1/VEGFR-2 receptor. The latter binds to VEGFA and represents a primary driver of angiogenesis in development and healthy conditions. Phenotypes from animal models are consistent with a negative modulation of vasculogenesis/angiogenesis by FLT1 during development [3]. However, FLT1 can positively regulate angiogenesis in the context of various stress responses and diseases, including cancer. Unlike FLK1, the FLT1 gene can be up-regulated by hypoxia due to the presence of an HIF-1α binding site in the promoter [4]. Moreover, only FLT1can bind to VEGFB and Placental Growth Factor (PlGF) ligands that are overexpressed in pathological conditions and result in activation of intracellular signals [3], [5]. The efficiency of signal transduction via FLT1 and upon binding to specific ligands can also be enhanced by the cell surface coreceptors neuropilin [6], that in addition to binding the semaphorins during neuronal development can also bind selected VEGF subtypes.

Consistent with a role in pathological angiogenesis, FLT1 can be up-regulated in several tumor types, including prostate, breast, colon and non-small cell lung cancer, lung adenocarcinoma, hepatocellular carcinoma and glioblastoma [7]–[12]. Notably, targeted reduction of FLT1 activity can inhibit tumor cell growth [13]–[15]. FLT1 is also important in tissue-specific metastasis, since bone marrow-derived hematopoietic progenitor cells that express FLT1 appear to be required for the formation of the “pre-metastatic niche” [16].

Recently, we established that expression from the FLT1 promoter could be modulated in response to genotoxic stress by concomitant activation of the p53 tumor suppressor and Estrogen Receptors (ERs) [17]. Modest p53-dependent responsiveness of the promoter was related to the presence of a single nucleotide polymorphism (SNP) in the proximal FLT1 promoter [18]. The relatively rare C>T allele results in a ½ site p53 response element (RE) that is necessary but not sufficient for a substantial p53-dependent transcriptional effect. Subsequent investigations showed that ligand-activated Estrogen Receptor acting at a ½ site Estrogen Receptor response element (ERE) located upstream of the p53 RE was required for high level of p53-dependent responsiveness [17].

The master regulator and tumor suppressor p53, which is one of the most important and studied proteins in the cancer field, is a tetrameric (dimer of dimer proteins) sequence-specific transcription factor able to bind to two copies of a decameric sequence with the RRRCWWGYYY consensus (where R stands for a purine, W for A/T and Y for a pyrimidine) [19]. Recent results, including our own investigation based on functional or DNA binding assays in cell systems or cell extracts, established that maximal transactivation requires adjacent dimer binding sites [20]–[26]. A spacer of a few bases dramatically reduced transactivation. We also established that p53 can stimulate transcription, albeit at a reduced levels, from noncanonical response elements including ½ sites [26] (reviewed in [27]). Deviations from consensus are common among established p53 target sites resulting in a wide range of transactivation potentials. The same sequence-specific requirements that were shown to maximize the transactivation potential from full site REs appear to be valid for the ½ site REs [26]. The C>T SNP in the FLT1 promoter changes a critical mismatch in a p53 ½ site that prevents function into a consensus sequence that provides for weak p53 transactivation [17].

The ERs belong to a large superfamily of nuclear receptor transcription factors that interact with specific ligands to regulate a variety of cellular pathways [28]. The ligand-induced conformational change enables ER to modulate transcription from EREs directly and through recruitment of cofactors. The ERE consensus sequence comprises two inverted repeats of the GGTCA pentamer, although EREs often contain nonconsensus bases [29]–[31]. The repeats are typically separated by a 3 nt-based spacer [32], [33]. Many ligands can interact with ER proteins and can impact differentially the transactivation at specific EREs. Acting in cooperation with other transcription factors such as Sp1 [34], ERs can also modulate transcription from ½ site EREs.

Our previous results with the FLT1 promoter established transcriptional cooperation between p53 and ER. The noncanonical nature of the cognate response elements present in the promoter enabled strong responsiveness only upon concomitant activation of both transcription factors. In this study these findings have been expanded to an ER positive, p53 wt breast cancer-derived cell model in order to better understand the cooperative relationship between ER and p53 in *cis*-regulation of FLT1 expression. We found that ER levels, specific ligands and genotoxic stresses can greatly influence the coordinated regulation of expression of the FLT1-T allele. In a more general sense, these findings indicate that a noncanonical p53 RE can provide a wide opportunity for fine-tuning p53-dependent cellular responses and for integrating signaling-responses to complex environmental perturbations.

## Results

### An additional ERE ½ site contributes to p53/ER responsiveness of the FLT1-T promoter

**Figure 1:**
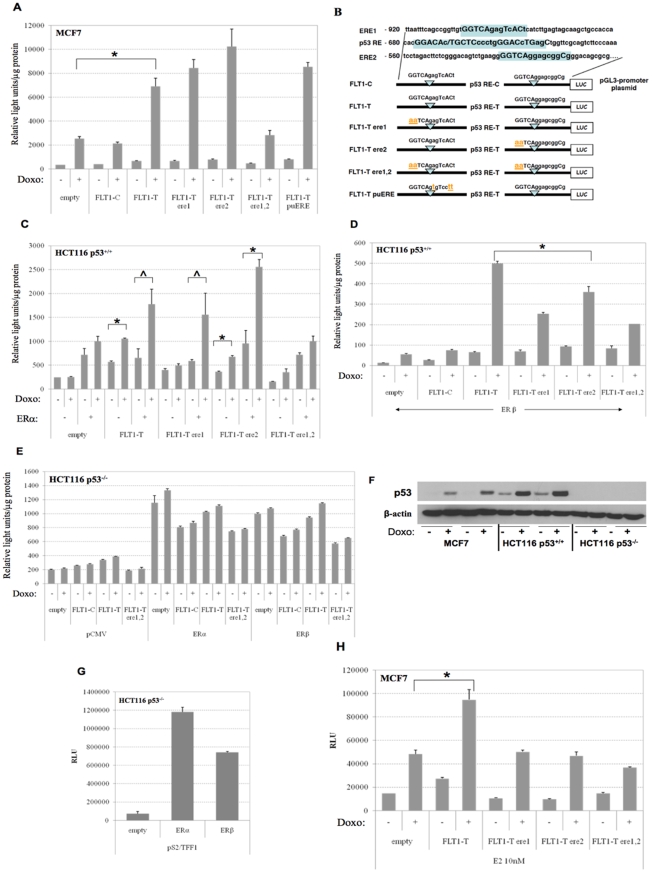
Two distinct ER ½ site response elements acting in cis along with estrogen receptors contribute to doxorubicin-induced, p53-dependent transactivation of the FLT1-T promoter. (A) MCF7 cells cultured in normal medium. (C & D). HCT116 p53^+/+^ cells. Gene reporter assays results are presented as the average light units and the standard errors of three independent biological replicates. The indicated reporter plasmids were transiently transfected along with the pRL-SV40 control vector. When indicated, the HCT116 p53^+/+^ cells were treated with doxorubicin (Doxo “+” ) at 0.3 µg/ml for 16 hours prior to cell lysis. Luciferase activity was measured 48 hours post-transfection and normalized for transfection efficiency. Statistically significant differences are highlighted (* =  p<0.01; ∧ = p<0.05, t-test). (B) Partial sequence of the 1 kb FLT1 proximal promoter. The ERE1, p53 RE and ERE2 sequences are highlighted (uppercase: bases matching the consensus sequences) and their relative position with respect to the Transcriptional Start Site (1) is shown. Also presented is a schematic drawing of the reporter constructs based in the pGL3 promoter vector containing a 1 Kb fragment of the FLT1 promoter. The specific mutations introduced to inactivate the ERE1 and ERE2 sites are indicated (bold lowercase). (C,D) Cells were transiently co-transfected with the empty pGL3 vector or the indicated reporter constructs along with an expression vector leading to overexpression of ERα (C) or ERβ (D). (E) HCT116 p53^−/−^ cells were used as a control for the p53-dependence of the combined effect of doxorubicin treatment and ERα or ERβ expression on the transactivation of the FLT1 promoter constructs. (F) Representative western blot showing the increase in p53 protein levels after doxorubicin treatment; β-actin was used as loading control. For each cell line, p53 protein levels were assessed in a duplicate experiment to confirm that the transfection with different reporter plasmids did not impact p53 stabilization. (G) HCT116 p53^−/−^ cells were transfected with the pS2/TFF1 ER reporter along with empty or overexpression vectors for ERα or ERβ. H. MCF7 cells cultured in estrogen-depleted media supplemented with 10 nM E2.

We previously established that in addition to the ½ site p53 RE (−677 from the Transcriptional Start Site, TSS) a ½ site ERE located 225 nt upstream of the p53 RE (referred to as ERE1; −902 from TSS) is required for efficient p53-induced transactivation of the FLT1 promoter [17]. The cooperative interaction was mainly studied in the colon adenocarcinoma HCT116 cell line that expresses wild-type p53 protein (referred to as p53^+/+^). This cell line is ERα negative and weakly expresses ERβ. To understand better the cooperativity between p53 and ERs in the regulation of FLT1 promoter and the response to genotoxic stress, we evaluated the role of ERs in the p53-dependent transactivation of the FLT1 using a clone of the p53 wild-type breast adenocarcinoma MCF7 cell line which is positive for ERα and has low ERβ expression. Surprisingly, while in HCT116 cells the disruption of the −902 ERE ½ site (*i.e.*, “ere1”) was sufficient to nearly abolish the transactivation from a 1 kb FLT1-T promoter construct [17], there was no impact of the disruption in MCF7 cells (Figure 1A). The FLT1-C promoter construct was equivalent to the empty vector.

To investigate the cell-specific impact, we first performed *in silico* analyses of the FLT1 promoter for transcription factor binding sites using the Genomatix MatInspector [35] as well as Transcription Element Search System (TESS) software (MatInspector: http://www.genomatix.de/products/MatInspector; TESS: University of Pennsylvania Computational Biology and Informatics Biology Laboratory, http://www.cbil.upenn.edu/tess). This led to the identification of a second putative ERE ½ site (ERE2: GGTCAggagcggC; mismatched based from a consensus full site ERE are underlined) located 145 nt downstream to the p53 RE (Figure 1B). To evaluate the contribution of ERE2 in the p53, ER mediated transactivation we developed luciferase-based reporter plasmids containing 1 kb FLT1-T promoter constructs harboring inactivating mutations at either of the ERE sites (“ere1” or “ere2”) as well as a double mutant (“ere1,2”) (described in details in Figure 1B). Gene reporter assays clearly showed that in MCF7 cells both EREs needed to be inactivated to impair p53-dependent transactivation (Figure 1A).

Next we asked whether the relative activity for ERE1 is strictly dependent on the ½ site context (GGTCA) or could be affected by the adjacent sequence 3′ to it (GGTCAgagTcACt ) which, unlike the case of ERE2, contains some features of an ERE ½ site in opposite orientation and correctly spaced from ERE1 although there are mismatches at two critical positions (lowercase) [30], [36]. We constructed a “pure” ERE1 ½ site by site-directed mutagenesis (referred to as “puERE”, where the remaining consensus ERE bases were changed -TcACt to Tcctt- see Table S1 and Figure 1B). This modification did not impact the induction of transactivation in MCF7 cells (Figure 1A).

We then addressed whether the difference in responsiveness seen between HCT116 [17] and MCF7 could be related to intracellular levels of ER proteins. Ectopic overexpression of either ERα or ERβ in HCT116 along with the various FLT1-T constructs and doxorubicin treatment resulted in all but the ere1,2 double mutant being responsive (Figure 1C and 1D). ERβ overexpression resulted in higher relative transactivation of FLT1-T in response to doxorubicin treatment, suggesting an important role for this receptor in the cooperation with p53. However, unlike the case of ERα overexpression, a partial defect associated with the individual ere1 and ere2 constructs was still apparent upon ectopic expression of ERβ. Notably, disruption of ERE2 alone did not completely abolish the responsiveness to doxorubicin even without ER ectopic expression, possibly related to the difference in sequence between ERE1 and ERE2 and their relative closeness to the ER consensus. Consistent with this, the responsiveness to doxorubicin of the puERE construct was significantly lower compared to FLT1-T in HCT116 cells (not shown). Surprisingly, the empty vector was inducible by doxorubicin when the ERs were overexpressed in HCT116 cells (especially with ERβ). An even higher induction of the empty vector by doxorubicin was observed in MCF7 cells (Figure 1A). This might in part be due to the presence in close proximity of both a p53 RE and an ERE in the pGL3 promoter plasmid as predicted by the Gemomatix MathInspector (not shown) and could thus be influenced by the expression of p53 and ER proteins. The p53-dependence of the doxorubicin treatment on transactivation of the FLT1-T constructs was confirmed with a p53-null HCT116-derived cell line (Figure 1E). In the absence of p53, the overexpression of either ERα or ERβ did not lead to any further differential stimulation of the FLT1 reporter constructs although a higher basal level with just the plasmid backbone was observed independent from the SNP status or the ERE sequences (Figure 1E). Doxorubicin treatment resulted in p53 protein stabilization in both p53 positive cell lines and was not affected by transfection with the reporter constructs (Figure 1F). Unlike the FLT1-T reporter, the pS2/TFF1 ER reporter construct was highly inducible by overexpressed ERα or ERβ in HCT116 p53^−/−^ cells, confirming the activity of the ER proteins in our culture conditions (Figure 1G). The relative impact of disrupting the ERE1 or ERE2 sites was evaluated in MCF7 cells also in estrogen-reduced culture conditions supplemented with 10 nM estradiol and treated or not with doxorubicin (Figure 1H). Interestingly, in these conditions only the disruption of one ERE was sufficient to bring the doxorubicin responsiveness down to the level of the empty vector. While the results confirmed that ERE2 is a functional ERE, they also highlighted the strong influence of the experimental conditions in the FLT1 cis-regulation.

**Figure 2:**
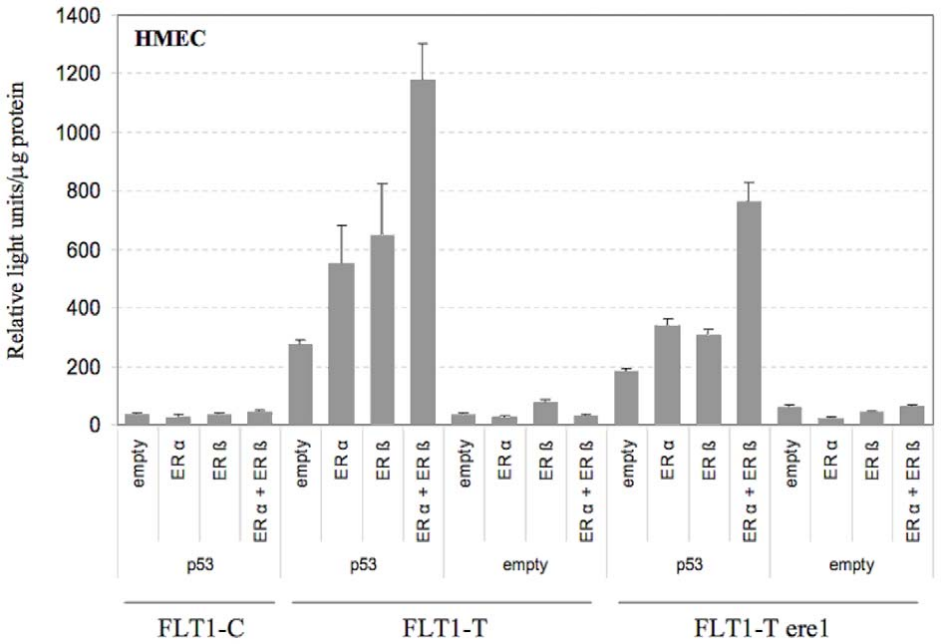
p53 and estrogen receptors cooperate in FLT1-T transactivation in HMEC cells. Cells were transiently co-transfected with the FLT1-C, FLT1-T or FLT1-T-ere1 reporter constructs along with expression vectors for p53, ERα and/or ERβ. Relative luciferase activity was determined 24 hours after transfection. Presented are the average relative light units and the standard errors of three biological repeats.

The transcriptional cooperation between p53 and ERs was also examined in human endothelial cells derived from dermal microvessels (HMEC) using ectopic expression conditions (Figure 2). While the FLT1-C construct was not responsive to p53 and/or ERs, transcriptional cooperation was observed with the FLT1-T, particularly when both ERα and ERβ were overexpressed together with p53. Consistent with the results in MCF7 and in HCT116 p53^+/+^ cells, the FLT1-T-ere1 construct was also inducible by ectopic expression of p53, especially when ERα and ERβ were also expressed ectopically.

### Neither p63 nor p73 can modulate transactivation of the FLT1 promoter construct

Since the p53-related transcription factors p63 and p73 can modulate p53 functions and activate the transcription of some p53 target genes [37], [38], we examined the impact at the FLT1 promoter alleles using gene reporter assays and ectopic expression of p53 or of selected p63 and p73 isoforms. We also evaluated the effect of doxorubicin treatment along with the ectopic over-expression.

**Figure 3:**
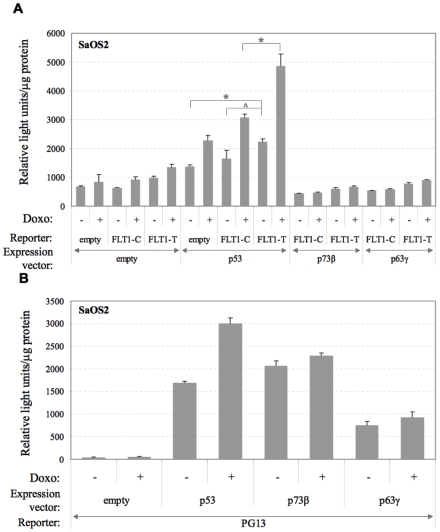
The p53-related p73β and p63γ proteins do not transactivate the FLT1 promoter constructs in SaOS2 cells. To avoid any contribution of the endogenous p53 protein, gene reporter assays were performed in a p53-null, osteosarcoma-derived SaOS2 cell line. (A) Cells were transiently co-transfected with the FLT1-C or FLT1-T reporter constructs along with an overexpression vector for p53, p73β or p63γ as well as the pRL-SV40 control vector, followed by doxorubicin (0.3 µg/ml) or mock treatment, according to the schedule described in Figure 1. Presented are the average-fold luciferase induction relative to FLT1-C mock treated and the standard errors of three replicates. Statistically significant differences relating to the impact of p53 expression alone or in combination with doxorubicin treatment are highlighted (* =  p<0.01; ^∧^ = p<0.05, t-test). (B) The PG13 p53 reporter plasmid was used as a control for comparing the transactivation potential of p53, p73β or p63γ and the effect of the doxorubicin treatment.

These experiments were conducted in SaOS2 cells which lack endogenous p53. Ectopic p53 expression resulted in modest transactivation of FLT1-T, consistent with the small amount of p53 expression plasmid that was used for transfections (100 ng/well). While in previous experiments more p53 plasmid was used (500 ng/well) [18], we chose conditions in which the impact of doxorubicin along with ectopic p53 expression could be assessed. In the absence of p53 the FLT1 promoter constructs exhibited low level transactivation similar to the empty vector (Figure 3A). The treatment with doxorubicin led to a small induction (<1.5 fold). The ectopic expression of p53 resulted in a weak stimulation for all reporter constructs (2.0, 2.6 and 2.3 fold, respectively, for empty vector, FLT-C and FLT1-T). The promoter context had an impact in p53-expressing cells, as FLT1-T but not FLT1-C resulted in significantly higher activity compared to the empty vector (1.6 *vs* 1.2 fold induction). Doxorubicin treatment in p53-expressing cells led to slightly higher transactivation of the FLT1-C reporter (1.3 fold) and greater transactivation of FLT1-T (2.1 fold). The impact of the SNP on the combination of ectopic p53 expression and doxorubicin treatment was highly significant. The PG13 reporter plasmid, a canonical p53 responsive construct, containing a tandem repeat of a p53 RE [39], exhibited high responsiveness to the ectopic expression of p53 that was further stimulated by the doxorubicin treatment (Figure 3B). Ectopic expression of the p53-related transcription factors p73β or p63γ (corresponding to the spliced isoforms that are transcriptionally more active) failed to modulate the response from the FLT1 promoter, regardless of SNP status, although the PG13 control reporter was induced by both proteins.

### Differential impact of doxorubicin and 5-fluorouracil on p53-dependent FLT1-T transactivation

**Figure 4:**
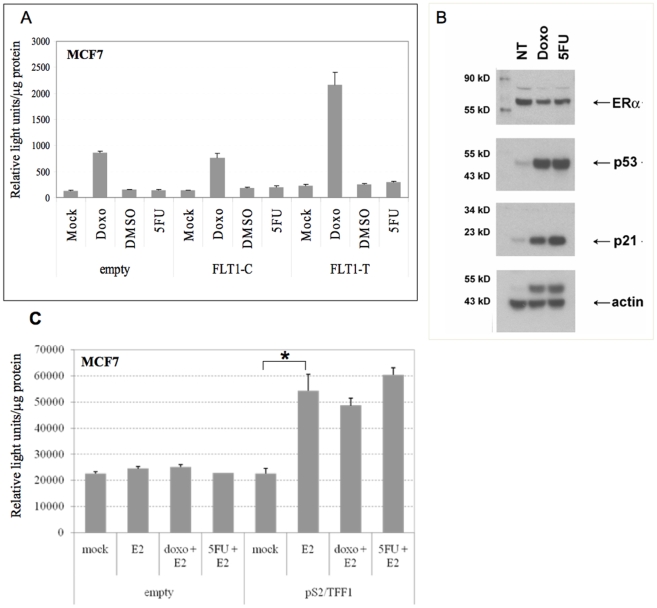
Differential effect of doxorubicin and 5-fluorouracil treatment on FLT1-T transactivation. (A) MCF7 cells were seeded into 12-well plates and transfected either with empty, FLT1-C or FLT1-T plasmids, along with the pRLSV40 control vector. 24 hours after transfection cells were treated with doxorubicin (0.3 µg/ml) or 5FU (375 µM) or mock treated, as indicated. At 16-hour post-treatment, cells were lysed for the luciferase assays. The relative averages and standard errors of at least three independent replicates are presented. (B) Western blot analysis of the p53 (DO-1 antibody), p21 (C-19 antibody) and ERα (H-184 antibody) proteins after treatment with doxorubicin and 5FU. 30 ug of total proteins were loaded in each lane. Actin (C-11 antibody) was used as loading control. All antibodies were obtained from Santa Cruz. The bands above actin are due to a residual p53 signal caused by incomplete removal of the primary Ab against p53 while re-probing for actin. (C) MCF7 cells cultured in estrogen-depleted medium for 72 hrs, were seeded into 24-well plates and transfected with empty or pS2/TFF1 reporter vector. 24 hours after transfection cells were treated with doxorubicin (0.3 µg/ml) or 5FU (375 µM) and/or with the addition of 100 nM E2. At 16-hour post-treatment, cells were lysed for the luciferase assays. The relative averages and standard errors of at least three independent replicates are presented. E2 led to a significant induction (* =  p<0.01, t-test) of the pS2/TFF1 reporter that was not impacted by doxo or 5FU treatment.

Next we examined whether the nature of the genotoxic stress that leads to p53 stabilization and activation could impact FLT1-T transactivation using MCF7 cells. Interestingly, the thymidylate synthase inhibitor 5-fluorouracil (5FU), another chemotherapeutic agent commonly used to study p53-mediated responses [40]–[42] did not result in transactivation of the FLT1-T construct, although p53 protein levels as well as p21 were increased comparably by doxorubicin and 5FU with respect to mock treated cells (Figure 4A and 4B). In addition, 5FU treatment did not considerably affect the expression levels of ERα protein (Figure 4B), nor its activity, based on gene reporter assays conducted in estrogen-depleted medium supplemented with 100 nM E2 (Figure 4C). Furthermore, as described below, the endogenous FLT1 gene expression was inducible by doxorubicin but not 5FU.

### Differential impact of doxorubicin and 5FU treatment on the recruitment of p53 at the RE-T and the role of ERE1 and ERE2

**Figure 5:**
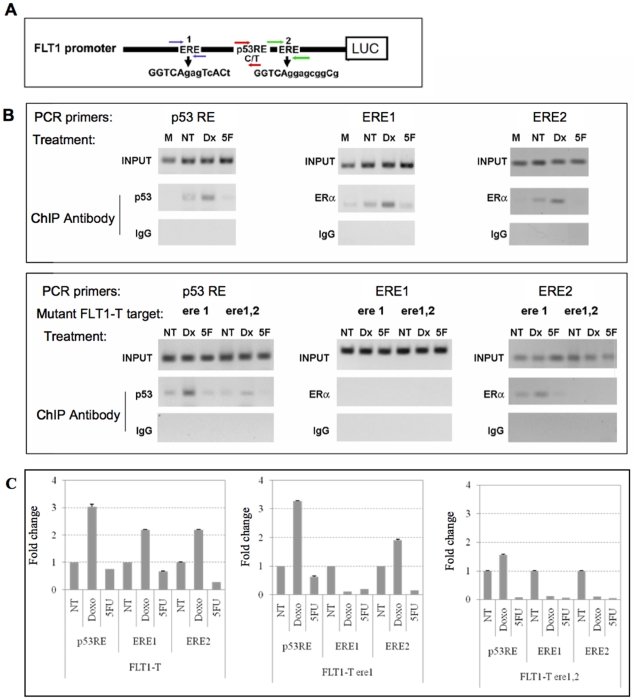
The ERE2 site is important in the recruitment of p53 and ERα on FLT1-T promoter constructs transfected in MCF7 cells. (A) Schematic drawing of the annealing positions of the PCR primers utilized in the ChIP experiments to probe the individual response elements. (B, top panels) representative PCR results that evaluate the occupancy of p53 or ERα after mock, doxorubicin and 5FU treatments obtained with primers specific for the p53 RE, ERE1 and ERE1 region. (B, lower panels) same as in top panel except that the indicated FLT1-T mutant constructs were transfected. ChIP PCR reactions for p53RE, ERE1 and ERE2 were performed with the single mutant ere1 or the double mutant ere1,2 within the pGL3 based constructs after ChIP with p53 and ERα antibodies, respectively. PCR results obtained with input DNA and with IP material obtained from a negative control IgG antibody are also shown. (C) Fold change in site occupancy measured using real time PCR. Data are presented following the same order as in (A). The antibody used for the ChIP experiment was targeted at p53 for the reported analysis at the p53RE site and at ERα for the analysis at the ERE1 or ERE2 sites. Bars represent the average fold change in occupancy and standard deviations of three technical replicates.

We previously demonstrated using HCT116 p53^+/+^ and p53-transfected SaOS2 cells that the ERα or ERβ could bind only the FLT1-T promoter construct in the presence of p53 suggesting that p53 binding was required for ER binding [17]. These findings have been extended to the impact of ERE1 and of the double ERE1, ERE2 mutations on occupancy at EREs and p53 REs using MCF7 cells transfected with the various FLT1 promoter constructs. Specific primers for amplifying the ERE1, ERE2 and the p53 RE sites on plasmid reporters were developed (Figure 5A and Table S2), as well as sonication conditions that enabled us to evaluate the distinct contribution of ERE1 and ERE2. The impact of doxorubicin and 5FU treatments were also compared (Figure 5). First we tested the ability of p53 and ERα to be recruited to a canonical target promoter site (p21 and TFF1, respectively). The different DNA damaging agents resulted in enhanced p53 occupancy at the p21 promoter, consistent with other reports in the literature [43], while those same treatments appeared to similarly reduce ER occupancy at the pS2 control target (Figure S1A and S1B). While doxorubicin-treatment led to a significant increase in p53 occupancy levels at the p53 RE-T within the FLT1 promoter fragment, treatment with 5FU did not induce p53 occupancy at the site, consistent with the failure of the 5FU-dependent FLT1-T transactivation (Figure 5B & 5C). Doxorubicin treatment also resulted in ERα occupancy detectable with the primers both for the ERE1 and ERE2 sites (Figure 5B, top panel; 5C, left panel). Inactivation of the ERE1 site did not affect p53 occupancy, but the inactivation of both ERE1 and ERE2 greatly reduced p53 occupancy, consistent with a cooperative *cis* interaction between p53 and ER (Figure 5B, lower panel; 5C center and right panel). Mutation of the ERE1 site abolished ERα recruitment at the site, but doxorubicin-enhanced occupancy was still detectable at the ERE2 site. The double ere1,2 mutation completely abrogated ERα occupancy. Overall, these results support a distinct role for p53-inducing cellular treatment that appears related to the specific nature of the *cis-*element sequences in FLT1. Furthermore they indicate that ERE2 is an active ER ½ site and suggest a contribution of ER proteins on the stability/recruitment of p53 at the promoter.

### ER ligands differentially impact FLT1-T transactivation

**Figure 6:**
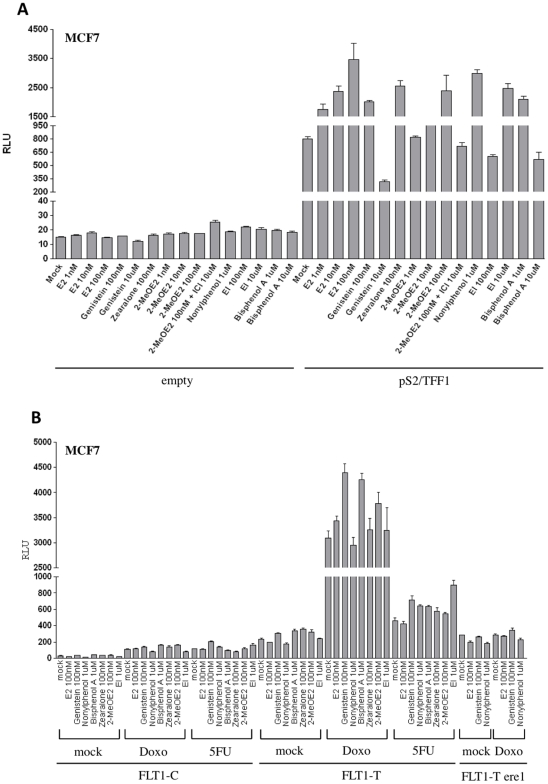
ER ligand-specific effects on FLT1-T transactivation in doxorubicin treated MCF7 cells. Cells were cultured in estrogen-depleted medium and serum, seeded into 12-well plates and co-transfected with the empty luciferase vector, the pS2/TFF1 ER reporter vector (A) or FLT1-C, FLT1-T or FLT1-Tere1 constructs (B) along with the pRL-SV40 control vector. Cells were incubated with the various ER ligands at the indicated concentrations. Where indicated, cells were also treated with doxorubicin (0.3 µg/ml) or 5FU (375 µM). Luciferase assays were performed on total protein extracts prepared 48 hours post-transfection (16 hours after DNA-damaging treatment, 10 hours after ligand addition). The histograms represent the average light units and the standard errors of three independent biological replicates. E2 = 17-β-estradiol; 2-MeO-E2  = 2-methoxyestradiol; EI = 2-methoxyestrone.

The ER activity at FLT1-T [17] is ligand dependent. We determined if ER ligands differ in their impact on FLT1-T transactivation. Using luciferase assays, the transiently transfected MCF7 cells were treated with several compounds chosen for their established or proposed estrogen-like activity along with doxorubicin. The responses with the pS2/TFF1 ER target promoter were compared to those on FLT1-T (Figure 6A and 6B). Doses were chosen that had similar effects with the pS2/TFF1 ER target promoter. Contrary to the effect on TFF1, genistein, 2-methoxyestradiol and bisphenol A appeared to be more effective than 17β-estradiol (E2) in enhancing doxorubicin-induced FLT1-T transactivation. 2-methoxyestradiol (2Me-OE2) was used based on a previous report of an agonist effect in MCF7 [44], [45]. Other reports have instead concluded that this molecule is not capable to engage the ERs [46]. We used different concentration of 2ME-OE2 and examined the impact on the pS2/TFF1 reporter. Interestingly, unlike E2 which was active at all concentrations tested (1, 10, 100 nM), 2Me-OE2 led to reporter transactivation only at the 100 nM concentration. This agonist effect was abolished by the addition of the ICI-182,780 ER antagonist. Zearalenone had a similar impact relative to E2 on the two reporters while 2-methoxyextrone (EI) had a little impact on FLT1-T and nonylphenol was inactive. None of the ligands had any impact on FLT1-T transactivation in the absence of doxorubicin treatment confirming our previous results that FLT1 promoter is not responsive to the ERs alone [17]. The FLT1-T construct was also not responsive to 5FU (less than two fold compared with a 13 fold induction by doxorubicin) (Figure 6B). Consistent with the results presented in Figure 1H, disruption of the ERE1 site abolished doxo responsiveness in estrogen depleted medium. Addition of 100 nM E2 or Genistein did not lead to any induction. The FLT1-C construct was not responsive. The induction observed after genotoxic treatment was equivalent with the empty vector (data not shown; see Figure 1). [17]

### p53-activating agents differentially impact the endogenous FLT1 gene expression

**Figure 7:**
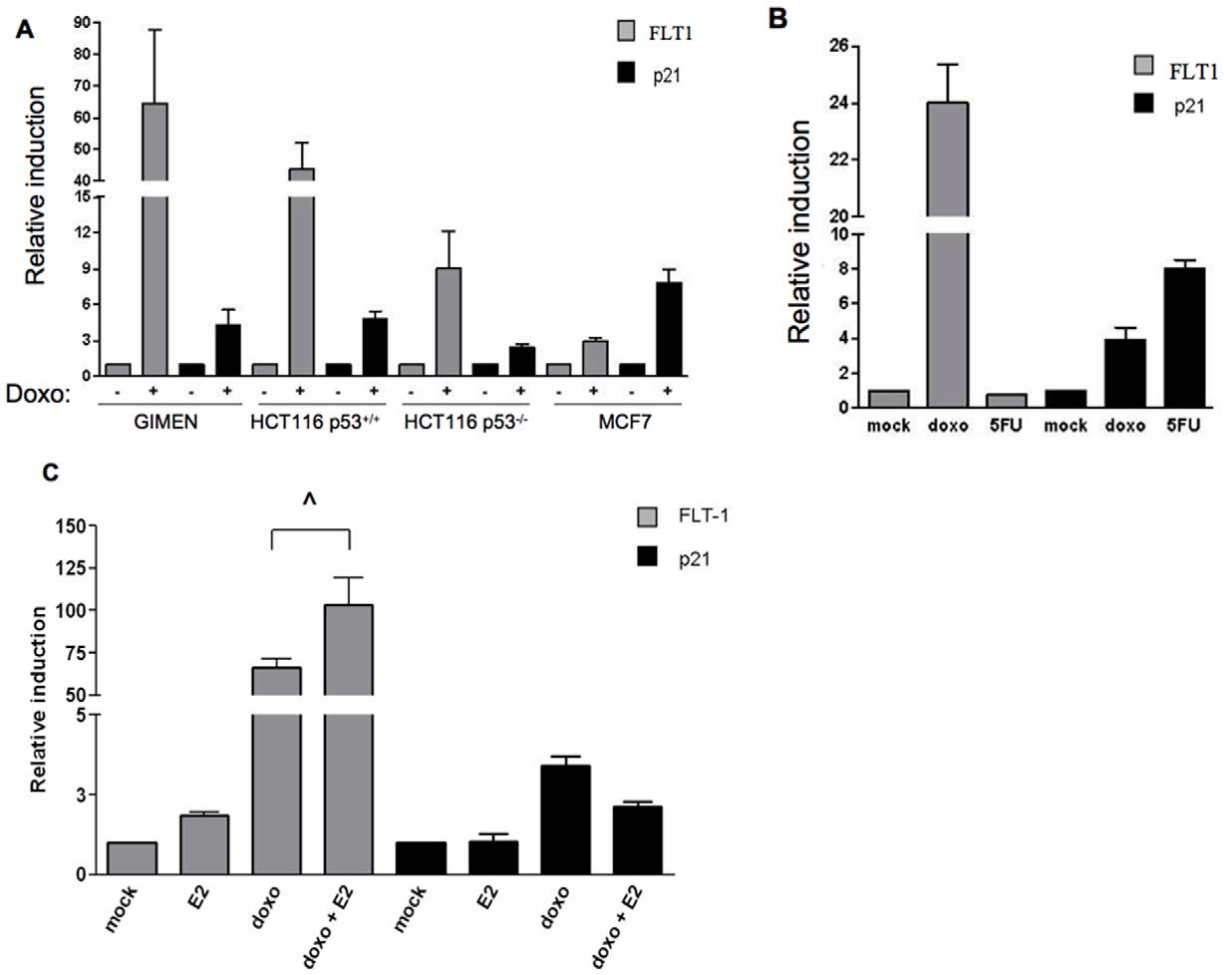
Exposure-dependent, differential induction of the endogenous FLT1 and p21 genes. The relative expressions of FLT1 (gray bars) and p53 target gene p21 (black bars) in mock, doxorubicin or 5FU treated cells were compared using an RT-PCR approach in real time. GIMEN (neuroblastoma-derived) and HCT116 (colon adenocarcinoma) are heterozygous (CT) for the promoter SNP at the FLT1 promoter while MCF7 cells are homozygous for the p53 nonresponsive SNP allele (CC). The HCT116 p53^−/−^ derivative cell line was used as control for p53-independent effects. GAPDH or B2M expression levels served as normalization controls. Histograms described the average fold-induction relative to the housekeeping gene, calculated using the ΔC_t_ method. Error bars correspond to standard errors of at least three replicates. (A) Comparison between GIMEN, HCT116 and MCF7 cells. (B) Differential impact of doxorubicin and 5FU on FLT1 expression in GIMEN cells. Since the basal levels of FLT1 expression were very low (C_t_≥30), the fold of induction may appear somewhat exaggerated. C) Impact of doxorubicin and/or E2 on FLT1 and p21 mRNA expression in GIMEN cells culture in estrogen-depleted medium. The increase in FLT1 mRNA expression by combined doxorubicin and E2 treatment appeared to be statistically significant (^∧^ = p<0.05, t-test).

Using real time RT-PCR approach we quantified endogenous mRNA expression levels of the *FLT1* gene after different genotoxic treatments. The p53 positive HCT116 p53^+/+^ and neuroblastoma-derived GIMEN cell lines were used because they were found to be heterozygous for the C>T SNP in the FLT1 promoter. The MCF7 (C/C) cells were used as controls. The relative mRNA levels for the p53 target gene p21 were measured in comparison with those of FLT1. The basal levels of *FLT1* mRNA varied considerably among the cell lines before treatment and were nearly undetectable in GIMEN, very low in HCT116, but significantly higher in MCF7 cells (data not shown). The *FLT1* mRNA was strongly induced by doxorubicin in GIMEN and HCT116, but not in MCF7 cells, while p21 was clearly induced in all three (Figure 7A). Transfected FLT1-T reporter constructs were inducible in the MCF7 cells (Figure 1A), demonstrating the potential for combined p53/ER-mediated regulation. Treatment of GIMEN with 5FU led to the induction of p21 expression but *FLT1* mRNA levels were not changed, consistent with the results obtained in the gene reporter assays (Figure 7B). To study at the endogenous gene level the cooperation between doxorubicin and E2, mRNA was quantified in GIMEN cells culture in estrogen-depleted medium (Figure 7C). Combined treatment with doxorubicin and E2 led to a significant increase in FLT1 mRNA expression compared the effect of doxorubicin alone. On the contrary the combined treatment slightly reduced p21 expression compared to doxorubicin alone. As an additional control we conducted real time RT-PCR analysis of HMEC cells that are homozygous (C/C) for the SNP in the FLT1 promoter. p53 and/or ERα and/or ERβ were ectopically expressed in these cells that were also treated with ER ligands (estradiol and diethylstilbestrol) or with an ER antagonist (ICI 182,780). Unlike the results with the FLT1-T reporter constructs (Figure 2), we did not observe any induction of the endogenous FLT1 gene, confirming that the combined p53/ER responsiveness requires the T allele and that the gene is not inducible by ERs alone (Figure S2).

## Discussion

We have characterized the in *cis* interaction between p53 and estrogen receptors that can result in transcriptional modulation of the FLT1 promoter containing a p53 responsive SNP in a breast cancer cell model where estrogen responses might be expected to be more significant. Specifically, we investigated *cis*-acting FLT1 promoter features and explored environmental components, namely genotoxic stress conditions and estrogenic ligand exposure, resulting in the combined activation of p53 and ERs. Importantly, we identified a second functional *cis*-acting ERE ½ site located 145 nt downstream to the p53 ½ site in the FLT1 promoter. Using site-specific mutagenesis, ectopic expression of ERs, transactivation assays and ChIP studies with the breast cancer-derived MCF7 cells, the colon cancer-derived HCT116 cells and the HMEC cells, we confirmed that this ERE2 can participate in the cooperative interaction between p53 and ERs. The ERE2 can recruit ERα even when the ERE1 sequence is mutated. Disruption of both EREs abrogated ER binding and nearly abolished p53 binding to the p53 RE-T region, consistent with the results of the functional assays. Notably, gene reporter assays, RT-qPCR for the endogenous gene, Western Blot and ChIP experiments consistently indicated that the nature of the genotoxic-stress resulting in p53 activation (*i.e.*, doxorubicin vs. 5-fluorouracil) dictated the engagement and the function of p53 at the FLT1 promoter.

The exact mechanisms that differentiate the impact of doxorubicin and 5FU on FLT1, including the involvement of other *cis*-acting sequences, remains to be explored and might be related to distinct p53 post-translational modifications induced by the signaling pathways activated upon treatment with these genotoxic drugs [41], [47]. Alternatively, the mechanisms may relate to the availability of cofactors or even the combined activation of other sequence-specific transcription factors, as proposed in previous studies designed to interpret stress-dependent differences in the global p53 transcriptional network [20], [48], [49], or may be a feature that is unique to a ½ site noncanonical p53 target. Overall, our results indicate that the noncanonical nature of the *cis*-acting elements that enables p53 and ER cooperation at the FLT1 promoter provides higher potential for adaptive tuning of the transcriptional responses compared to highly responsive p53 target genes, such as p21, and may suggest differences in responsiveness to altered function p53 mutants [27].

In a recent study [26] we examined p53 transactivation potential using various permutations of ½ site RE sequences, confirming that p53 transactivation from this type of noncanonical regulatory sequence is affected by specific sequence features of the RE, similar to what is observed for full-site (tetrameric) canonical REs. The FLT1 ½ site (GGACATGCTC) ranked low in the comparison of the transactivation potential of ½ site REs, despite the presence of the CATG in the core sequence. In fact, we noted an unexpected negative impact on transactivation that was associated with the “CT” sequence context in the RE. When a transcriptionally optimized ½ site was used, there was a ∼20 fold higher transactivation potential compared to the FLT1 RE [26]. It will be important to determine the effect of varying the intrinsic transactivation potential of a p53 RE on the combinatorial interaction between p53 and the estrogen receptor.

In this work, we also explored the contribution of the p53-related p63 and p73 transcription factors in the transactivation of FLT1 and the cooperation with ER, drawing upon recent reports on the differential DNA binding specificity between p53 and p63 [50], [51]. The reported preference of p63 for an “S” base (*i.e.* a C or G) instead of a “W” in the core p53 RE (CWWG) suggested to us the intriguing possibility that p63 and p73 may exhibit preferential activity towards the FLT1-C allele. However, experiments in SaOS2 cells indicated that neither p63 nor p73 play a role in FLT1 transactivation, regardless of SNP status. This lack of activity could be related to a generally lower DNA binding/transactivation potential of p63 and p73 towards weak p53 REs, consistent with previous observations [52], or to the fact that the cooperativity with the ERs is achieved by specific cofactors whose interaction with the p53 family of transcription factors is restricted to the p53 protein.

In this study we confirmed that the effect of ER on FLT1 transactivation requires at least one *cis-*acting ERE and is dependent on the interaction with specific ligands. ERα and ERβ appeared to act similarly in the in *cis* interaction with p53 [17], although we noted a stronger impact of ERβ in HCT116 cells. The disruption of a single ERE did not impact doxorubicin responsiveness in the MCF7 cells (Figure 1A) and had a small effect in HCT116 p53+/+ or HMEC cells when ERα and especially ERβ were overexpressed (Figure 1C, D and Figure 2). Surprisingly, when MCF7 cells were cultured in estrogen-reduced medium supplemented with 10 nM E2, disruption of either ERE1 or ERE2 resulted in loss of doxorubicin responsiveness (Figure 1H). Consistent with this, previous experiments developed in the osteosarcoma derived U2OS and SaOS2 cells, showed that disruption of a single ERE (ere1) impaired p53-dependent transactivation even with ectopic expression of ERα or ERβ [17]. ChIP assays in SaOS2 showed that ERE1 disruption did not impair p53 binding at the p53 RE but seemed to affect ER binding [17], based on the lack of recruitment of the TRA220 subunit of the mediator complex that can bind to ERs [53]. Using MCF7 cells, in this study we have confirmed that p53 binding is required for ER binding, but have also revealed that ERα occupancy can stabilize p53 binding at FLT1-T given that disruption of both EREs strongly reduced p53 occupancy. Taken collectively, our results suggest that, besides ER protein levels, other factors can modulate the p53/ER cooperation at the FLT1 proximal promoter in the context of a doxorubicin response. This is consistent with the genotoxic stress-dependency previously described and the cell type specific effects.

Given that only ½ site EREs are present in the FLT1 promoter and that p53 is required for ER-dependent transcriptional stimulation, we evaluated the impact of different ER ligands on FLT1-T transactivation and compared responses with a typical estrogen-responsive promoter, derived from the pS2/TFF1 gene using MCF7 cells cultured in estrogen-reduced conditions. Previous studies revealed ligand dependencies in target specificity [30]. Included in our test were the dietary phytoestrogens genistein and zearalenone and the industrial estrogens *bisphenol* A and nonylphenol [30]. We also tested 2-methoxyestradiol, that was shown to exhibit anti-angiogenic activity and could have agonist activity on ERs at high concentrations [45] and 2-methoxyestrone another estrogen metabolite that had been investigated for anti-proliferative properties [44]. Ligand doses were chosen based on the literature [30] and on assays with the pS2/TFF1construct (Figure 6A). While exploratory in nature, our results suggest a differential impact of the various ligands in the FLT1-T promoter context, as compared to pS2/TFF1. In particular, genistein and bisphenol A appeared more active than 17β–estradiol, while nonylphenol and 2-methoxyestrone were not active (Figure 6B). It will be interesting to address the relative impact in the FLT1 promoter motif where the ½ site is replaced by a full-site. Previous studies compared different ER ligands using reporter constructs with different ERE sequences [30], [31]. While promoter specific effects were reported for the impact of specific ligands or the relative contribution of ERα and ERβ, the quantitative differences among the impacts of genistein, bisphenol A and nonylphenol relative to 17β–estradiol were smaller compared to what is seen with the FLT1-T promoter construct.

The value of p53 mutant status as an independent tumor prognostic marker was found in large cohorts of sporadic breast cancers [54]. Recently, immunohistochemical and molecular analyses have identified different breast cancer subtypes [55], [56]. Among these, the basal-like tumors, clinically very aggressive, are defined by the lack of expression of ER, Progesterone Receptor (PR) as well as epidermal growth factor receptor 2 (HER2) and by a high frequency of p53 mutations. Instead the luminal subtypes are p53 wild type and ER/PR positive and associated with a more favorable prognosis. However, luminal B subtype tumors that are classified based on the reduction or loss of ER/PR expression, in some cases associated with gain of HER2 expression, have a significantly worse outcome (54). Direct molecular links between ER expression, p53 WT or mutant status, and disease outcome still need to be fully established. The transcriptional cooperation we uncovered at FLT1 should be evaluated in this context.

The transcriptional circuit provided by the C>T SNP at the FLT1 promoter, appears to bring an additional angiogenesis gene into the p53 transcriptional network. Previous studies have linked p53 to the modulation of angiogenesis both through transcriptional repression of VEGF [57] and induction of the metalloproteinase MMP2 [58], maspin [59] and PAI [60]. p53 was also shown to be directly involved in the degradation of collagen, resulting in the production of antiangiogenic peptides [61]. Other studies have linked p53 to the control of the migratory potential of various cell types, including macrophages [62], [63]. Importantly, ER proteins have also been directly linked to the up-regulation of VEGF [64]. Recent results in the literature have highlighted the potential key role of membrane-bound FLT1 in pathological angiogenesis, especially in the context of cancer growth and spread [3]. In particular, FLT1 is the main receptor for VEGFB and PlGF induced signaling and is over-expressed in several cancer cells [3]. In the case of neuroblastoma, FLT1 expression was linked to chemoresistance, especially in the context of low oxygen tension [8]. Furthermore, bone-marrow-derived FLT1-positive cells are required for the initial formation of pre-metastatic niches in a model of lung tissue metastasis in nude mice [16]. The biological consequences of the inclusion of FLT1 among the p53 target genes are, however, difficult to predict and await specific investigations. Furthermore, the complexity of the FLT1 regulation is enhanced by the synthesis of a soluble form of the receptor (sFLT1), deriving from alternative splicing of the FLT1 primary transcript that can exert opposite functions compared to the membrane-bound, full-length form (mFLT1) [65]–[67].

Overall, our results have identified *cis-* and *trans-* factors resulting in the integration of signaling responses that coordinate allele-specific FLT1 transactivation by p53 and estrogen receptors. This study provides further evidence for the functional role of p53 ½ site response elements in modulating p53 transcriptional responses resulting in concerted regulation of gene targets through cooperation with other sequence-specific TFs. This type of functional interaction could be relevant for the modulation of many yet undiscovered gene targets and contribute to the regulation of p53 transactivation selectivity, tailoring responses to specific cellular stress conditions. In a related study, we have examined the influence of the FLT1 promoter motif on the p53/ER transcriptional cooperation with various p53 response element sequences—both canonical and noncanonical placed within the FLT1 promoter context--. This study (in press) has revealed the generality of the *in cis* interaction between p53 and ER; furthermore, we showed that the enhanced transactivation can extend to cancer-associated p53 mutations.

## Materials and Methods

### Cell lines and culture conditions

The human breast adenocarcinoma-derived MCF7 cell line (p53 wild type, positive for both ERα and ERβ) was obtained from the InterLab Cell Line Collection bank, ICLC (Genoa, Italy). The metastatic neuroblastoma GIMEN cells (p53 wild type) were obtained from GP Tonini (National Institute for Cancer Research, IST, Genoa, Italy), while the colon adenocarcinoma HCT116 (p53^+/+^) cell line and its p53^−/−^ derivative and the osteosarcoma-derived SaOS2 cells (p53-null) were a gift from B. Vogelstein (The Johns Hopkins Kimmel Cancer Center, Baltimore, Maryland, USA). Human endothelial cells from dermal microvessels were derived in the Schoenfelder lab and cultured as previously described. Cells were normally maintained in DMEM or RPMI supplemented with 10% FCS and antibiotics (100 units/ml penicillin plus 100 µg/ml streptomycin). GIMEN medium was supplemented with +1% non-essential amino acids. For the experiments with the ER ligands, cells were instead maintained in estrogen-depleted medium consisting of DMEM or RPMI without Phenol Red (Euroclone, Celbio, Milan, Italy) supplemented with 10% Charcoal-Dextran treated FCS (Hyclone, Celbio, Milan, Italy).

### Plasmids

Vectors carrying 1 Kb fragments derived from the human FLT1 proximal promoter were cloned in pGL3 promoter backbone (Promega, Milan, Italy) as described previously [17]. FLT1-T refers to the fragment containing the rare C>T SNP. FLT1-C is an equivalent fragment containing the common C allele. Site-directed mutagenesis (Stratagene, Milan, Italy) or a two-round, PCR-based, site-specific mutagenesis approach were performed to mutate the putative Estrogen Receptor Response Elements (EREs) within the FLT1-T promoter construct. The sequences of the primers used for site-specific mutagenesis are presented in Table S1. Mammalian expression plasmids harboring p63 and p73 cDNAs were a gift from G. Blandino (Regina Elena Cancer Institute, Rome, Italy) and M. Levrero (University of Rome “La Sapienza”, Rome, Italy). The PG13 p53 reporter plasmid (obtained from B. Vogelstein, The Johns Hopkins Kimmel Cancer Center, Baltimore, Maryland, USA) [39], was used as a transactivation control. The pS2/TFF1 reporter vector contains 1.3 kb of the proximal promoter of the estrogen-responsive gene TFF1 cloned in the pGL3-basic backbone [68]. All mammalian constructs were extracted from XL1blue *E. coli* cells using QIAex endofree maxi prep kit, according to the manufacturer's protocol (QIAGEN, Milan, Italy).

### Luciferase Assays in Transient Transfection Experiments

For each cell line 1-2.5×10^5^ or 4-8×10^4^ cells were seeded respectively in 12-well plates or 24- well 24 h before the transfection. Cells were transfected using FuGENE 6 (Roche, Milan, Italy) according to manufacturer's instructions at ∼80% cell confluence. For both types of plates, 300 ng of the pGL3-FLT1 reporter plasmids or pGL3-TFF1/pS2 ER reporter or PG13 p53 reporter were used. When appropriate, 100 ng of the expression vector for ERα/ERβ or for p53 family members (or a control empty vector) were included. All transfections were normalized for efficiency using 50 ng of the pRLSV40 plasmid, harboring the luciferase gene from *Renilla reniformis* controlled by a constitutive promoter (Promega, Milan, Italy). The total plasmid DNA amount per well was kept constant at 450 ng adding the empty vector pCMV-NeoBam, when appropriate. 24 h after transfection doxorubicin or 5-fluorouracil (5FU) were added in the medium at the indicated doses. In all experiments, cells were harvested 16 h after drug treatment and luciferase assays were conducted as described previously [68].

### Western Blot Analysis

Cell extracts were quantified using the BCA protein assay kit and BSA as a reference standard (Pierce, Celbio, Milan, Italy), and equal amount of proteins were resolved on 7.5% BisTris Acrilamide gels, and transferred to Nitrocellulose or PVDF membranes (GE Healthcare, Milan, Italy) using a Biorad MiniProtean apparatus (Biorad, Milan, Italy). After blotting the membranes were probed with monoclonal (p53 specific: pAb1801 and DO-1, Santa Cruz Biotechnology, Milan, Italy) or polyclonal antibodies (p53: CM-1, Novocastra, Milan, Italy; p21: C-19; ERα: H-184, Santa Cruz Biotechnology, Milan, Italy). The quality as well as the equal loading and transfer of protein blots were determined by Ponceau S staining or using a monoclonal antibody against β-actin (C-11 Santa Cruz Biotechnology, Milan, Italy). The relative Molecular mass (*M*_r_) values of the immunoreactive bands were determined by using molecular weight markers (Fermentas, Milan, Italy). After washing, blots were incubated with the appropriate IgG- horseradish peroxidase conjugated secondary antibody (Santa Cruz Biotechnology, Milan, Italy), and immune complexes were visualized by using ECL plus reagent (GE Healthcare, Milan, Italy).

### Chromatin Immunoprecipitation (ChIP) Assays

ChIP assays were done as previously described [17] using the ChIP kit (Upstate Biotechnology, Millipore, Lake Placid, NY, USA). Briefly, cells were plated onto 150-mm dishes. After 24 h of treatment with doxorubicin or 5FU, cells were fixed with 1% formaldehyde for 10 min at 37°C and then treated with 125 mM glycine for 5 min. Samples were processed following the manufacturer's instructions. Cell lysates were then sonicated using conditions that enabled us to evaluate the distinct contribution of ERE1 and ERE2 which are less then 400 nt distant. The sonication was done using a Misonix 3000 instrument equipped with a deep cup horn. Samples were sonicated using six cycles of 20 second pulses at power setting 8 with a 40 seconds pause in-between. One microgram of ERα (H-184, Santa Cruz, Biotechnology) and DO-7 p53-specific monoclonal antibodies (BD Biosciences Pharmingen, San Jose, CA, USA) were used per ChIP assay. As a negative control we used mouse or rabbit Ig (Santa Cruz Biotechnology). PCR amplifications were performed on immunoprecipitated chromatin using primers to amplify specific regions in the FLT1 promoter (Table S2). The PCR cycles were as follows: an initial 10 min Taq Gold polymerase at 95°C followed by 40 cycles of 95°C for 15 s and 60°C for 1 min. The PCR products were then run on a 1.8% agarose gel and quantified with IMAGEQUANT V5.1 (Molecular Dynamics-GE, Piscataway, NJ, USA). Alternatively, qPCR in real time was used to quantify the fold change in site occupancy. qPCR reaction was done with 2 µL of each sample and using the Power SYBR® Green PCR Master Mix (Applied Biosystems, Foster City, CA, USA) following the manufacturer's recomendation. To determine the fold change in site occupancy the SuperArray ChIP-qPCR Data Analysis tool was used (SA Biosciences, Frederick, MD, USA).

### Real Time PCR

For the mRNA expression analyses, MCF7, HCT116 and GIMEN cell lines were seeded onto 100 mm Petri dishes and allowed to reach 70–80% of confluence before treating with different drugs as described in the figures. At least 16 hours after treatment cells were harvested and washed once with PBS. Instead, HMEC cells were cultured in estrogen-depleted medium, transiently transfected with p53 and/or ERs expression vectors and treated with pro- and anti- estrogenic compounds at different concentrations. Total RNA was extracted using the RNeasy Kit (Qiagen, Milan, Italy) according to the manufacturer's instructions. For real-time quantitative PCR, cDNA was generated from 1 µg of RNA by using the AffinityScript cDNA Synthesis Kit (Stratagene, Milan, Italy). Real-time PCR was performed on a RotorGene 3000 thermal cycler (Corbett Life Science, Ancona, Italy) using the 5PRIME MasterMix (Eppendorf, Milan, Italy). Primers and TaqMan probes are presented in Table S2. Relative mRNA quantification was obtained using the Δ_Ct_ method, where the Glyceraldehyde 3-phosphate dehydrogenase (GAPDH), the β_2_Microglobulin (B2M) or the β-actin genes served as internal control.

## Supporting Information

Figure S1 Impact of doxorubicin and 5FU on p53 and ER occupancy at target sites. (A): Doxorubicin and 5FU treatment result in a similar increase of p53 occupancy at the p21 promoter. (B) Doxorubicin and 5FU treatment showed a similar negative effect on ERα occupancy at the TFF1 promoter. Shown are representative PCR results obtained using template DNA retrieved from ChIP experiment conducted in MCF7 cells with the indicated primary antibodies and primers specific for the p53 RE and ERE1 containing regions of the target promoters. The effect of doxorubicin and 5FU were compared. In addition to a p53 specific Ab (DO1) and an ERα Ab (H-184 Santa Cruz) the IgG Ab was used as negative control. PCR of input DNA is also shown. (C) Fold change in site occupancy measured using real time PCR. Data are presented following the same order as in panel A. The antibody used for the ChIP experiment was targeted at p53 for the p21 promoter site and at ERα for pS2/TFF1 promoter site.
(0.21 MB DOC)

Figure S2 Quantification of FLT1 mRNA in response to p53, ER overexpression in HMEC cells. (A) Cells were transfected with expression vectors for p53, ERα or ERβ as indicated. (B) Cells co-transfected with p53, ERα and ERβ were also treated 24 hours after transfection by estrogen ligands (Estradiol, E2; Diethylstilbestrol, DES) at the indicated concentrations (M). When indicated, treatment included a 100-fold excess of the ER antagonist ICI 182,780. For both panels, histograms represent the average fold of induction relative to the beta-actin housekeeping gene, calculated using the ΔC_t_ method. Error bars present the standard errors of at least three replicates.
(0.09 MB DOC)

Table S1 Primers for 2-round PCR mutagenesis of 1 Kb FLT1-T constructs and description of method.
(0.05 MB DOC)

Table S2 List of the primers (A), the probes (B) used in the Real Time PCR experiments and (C) the primers used in ChIP experiments.
(0.05 MB DOC)

## References

[1] Ferrara N, Gerber HP, LeCouter J (2003). The biology of VEGF and its receptors.. Nat Med.

[2] Autiero M, Waltenberger J, Communi D, Kranz A, Moons, L (2003). Role of PlGF in the intra- and intermolecular cross talk between the VEGF receptors Flt1 and Flk1.. Nat Med.

[3] Fischer C, Mazzone M, Jonckx B, Carmeliet P (2008). FLT1 and its ligands VEGFB and PlGF: drug targets for anti-angiogenic therapy?. Nat Rev Cancer.

[4] Gerber HP, Condorelli F, Park J, Ferrara N (1997). Differential transcriptional regulation of the two vascular endothelial growth factor receptor genes. Flt-1, but not Flk-1/KDR, is up-regulated by hypoxia.. J Biol Chem.

[5] Luttun A, Tjwa M, Carmeliet P (2002). Placental growth factor (PlGF) and its receptor Flt-1 (VEGFR-1): novel therapeutic targets for angiogenic disorders.. Ann NY Acad Sci.

[6] Soker S, Takashima S, Miao HQ, Neufeld G, Klagsbrun M (1998). Neuropilin-1 is expressed by endothelial and tumor cells as an isoform-specific receptor for vascular endothelial growth factor.. Cell.

[7] Andre T, Kotelevets L, Vaillant JC, Coudray AM, Weber L (2000). Vegf, Vegf-B, Vegf-C and their receptors KDR, FLT-1 and FLT-4 during the neoplastic progression of human colonic mucosa.. Int J Cancer.

[8] Das B, Yeger H, Tsuchida R, Torkin R, Gee MF (2005). A hypoxia-driven vascular endothelial growth factor/Flt1 autocrine loop interacts with hypoxia-inducible factor-1alpha through mitogen-activated protein kinase/extracellular signal-regulated kinase 1/2 pathway in neuroblastoma.. Cancer Res.

[9] Fan F, Wey JS, McCarty MF, Belcheva A, Liu W (2005). Expression and function of vascular endothelial growth factor receptor-1 on human colorectal cancer cells.. Oncogene.

[10] Ghanem MA, van Steenbrugge GJ, Sudaryo MK, Mathoera RB, Nijman JM (2003). Expression and prognostic relevance of vascular endothelial growth factor (VEGF) and its receptor (FLT-1) in nephroblastoma.. J Clin Pathol.

[11] Ilhan N, Deveci F (2004). Functional significance of vascular endothelial growth factor and its receptor (receptor-1) in various lung cancer types.. Clin Biochem.

[12] Lee TH, Seng S, Sekine M, Hinton C, Fu Y (2007). Vascular endothelial growth factor mediates intracrine survival in human breast carcinoma cells through internally expressed VEGFR1/FLT1.. PLoS Med.

[13] Heidenreich R, Machein M, Nicolaus A, Hilbig A, Wild C (2004). Inhibition of solid tumor growth by gene transfer of VEGF receptor-1 mutants.. Int J Cancer.

[14] Ishizaki H, Tsunoda T, Wada S, Yamauchi M, Shibuya M (2006). Inhibition of tumor growth with antiangiogenic cancer vaccine using epitope peptides derived from human vascular endothelial growth factor receptor 1.. Clin Cancer Res.

[15] An P, Lei H, Zhang J, Song S, He L (2004). Suppression of tumor growth and metastasis by a VEGFR-1 antagonizing peptide identified from a phage display library.. Int J Cancer.

[16] Kaplan RN, Riba RR, Zacharoulis S, Bramley AH, Vincent L (2005). VEGFR1-positive haematopoietic bone marrow progenitors initialte the pre-metastatic niche.. Nature.

[17] Menendez D, Inga A, Snipe J, Krysiak O, Schonfelder G (2007). A single-nucleotide polymorphism in a half-binding site creates p53 and estrogen receptor control of vascular endothelial growth factor receptor 1.. Mol Cell Biol.

[18] Menendez D, Krysiak O, Inga A, Krysiak B, Resnick MA (2006). A SNP in the flt-1 promoter integrates the VEGF system into the p53 transcriptional network.. Proc Natl Acad Sci USA.

[19] el-Deiry WS, Kern SE, Pietenpol JA, Kinzler KW, Vogelstein B (1992). Definition of a consensus binding site for p53.. Nat Genet.

[20] Wei CL, Wu Q, Vega VB, Chiu KP, Ng P (2006). A global map of p53 transcription-factor binding sites in the human genome.. Cell.

[21] Tokino T, Thiagalingam S, el-Deiry WS, Waldman T, Kinzler KW (1994). p53 tagged sites from human genomic DNA.. Hum Mol Genet.

[22] Veprintsev DB, Fersht AR (2008). Algorithm for prediction of tumour suppressor p53 affinity for binding sites in DNA.. Nucleic Acids Res.

[23] Wang Y, Schwedes JF, Parks D, Mann K, Tegtmeyer P (1995). Interaction of p53 with its consensus DNA-binding site.. Mol Cell Biol.

[24] McLure KG, Lee PW (1998). How p53 binds DNA as a tetramer.. Embo J.

[25] Inga A, Storici F, Darden TA, Resnick MA (2002). Differential transactivation by the p53 transcription factor is highly dependent on p53 level and promoter target sequence.. Mol Cell Biol.

[26] Jordan JJ, Menendez D, Inga A, Nourredine M, Bell D (2008). Noncanonical DNA motifs as transactivation targets by wild type and mutant p53.. PLoS Genet.

[27] Menendez D, Inga A, Resnick MA (2009). The expanding universe of p53 targets.. Nat Rev Cancer.

[28] Deroo BJ, Korach KS (2006). Estrogen receptors and human disease.. J Clin Invest.

[29] Hall JM, Couse JF, Korach KS (2001). The multifaceted mechanisms of estradiol and estrogen receptor signaling.. J Biol Chem.

[30] Hall JM, Korach KS (2002). Analysis of the molecular mechanisms of human estrogen receptors alpha and beta reveals differential specificity in target promoter regulation by xenoestrogens.. J Biol Chem.

[31] Hall JM, McDonnell DP, Korach KS (2002). Allosteric regulation of estrogen receptor structure, function, and coactivator recruitment by different estrogen response elements.. Mol Endocrinol.

[32] Driscoll MD, Sathya G, Muyan M, Klinge CM, Hilf R (1998). Sequence requirements for estrogen receptor binding to estrogen response elements.. J Biol Chem.

[33] Klein-Hitpass L, Ryffel GU, Heitlinger E, Cato, AC (1988). A 13 bp palindrome is a functional estrogen responsive element and interacts specifically with estrogen receptor.. Nucleic Acids Res.

[34] Porter W, Saville B, Hoivik D, Safe S (1997). Functional synergy between the transcription factor Sp1 and the estrogen receptor.. Mol Endocrinol.

[35] Cartharius K, Frech K, Grote K, Klocke B, Haltmeier M (2005). MatInspector and beyond: promoter analysis based on transcription factor binding sites.. Bioinformatics.

[36] Gewirth DT, Sigler PB (1995). The basis for half-site specificity explored through a non-cognate steroid receptor-DNA complex.. Nat Struct Biol.

[37] Levrero M, De Laurenzi V, Costanzo A, Gong J, Wang JY (2000). The p53/p63/p73 family of transcription factors: overlapping and distinct functions.. J Cell Sci.

[38] Flores ER, Tsai KY, Crowley D, Sengupta S, Yang A (2002). p63 and p73 are required for p53-dependent apoptosis in response to DNA damage.. Nature.

[39] Kern SE, Pietenpol JA, Thiagalingam S, Seymour A, Kinzler KW (1992). Oncogenic forms of p53 inhibit p53-regulated gene expression.. Science.

[40] Sun XX, Dai MS, Lu H (2007). 5-fluorouracil activation of p53 involves an MDM2-ribosomal protein interaction.. J Biol Chem.

[41] Ju J, Schmitz JC, Song B, Kudo K, Chu E (2007). Regulation of p53 expression in response to 5-fluorouracil in human cancer RKO cells.. Clin Cancer Res.

[42] Kaeser MD, Iggo RD (2004). Promoter-specific p53-dependent histone acetylation following DNA damage.. Oncogene.

[43] Espinosa JM, Verdun RE, Emerson BM (2003). p53 functions through stress- and promoter-specific recruitment of transcription initiation components before and after DNA damage.. Mol Cell.

[44] Newman SP, Leese MP, Purohit A, James DR, Rennie CE (2004). Inhibition of in vitro angiogenesis by 2-methoxy- and 2-ethyl-estrogen sulfamates.. Int J Cancer.

[45] Sutherland TE, Schuliga M, Harris T, Eckhardt BL, Anderson RL (2005). 2-methoxyestradiol is an estrogen receptor agonist that supports tumor growth in murine xenograft models of breast cancer.. Clin Cancer Res.

[46] LaVallee TM, Zhan XH, Herbstritt CJ, Kough EC, Green SJ (2002). 2-Methoxyestradiol inhibits proliferation and induces apoptosis independently of estrogen receptors alpha and beta.. Cancer Res.

[47] Kaeser MD, Pebernard S, Iggo RD (2004). Regulation of p53 stability and function in HCT116 colon cancer cells.. J Biol Chem.

[48] Riley T, Sontag E, Chen P, Levine A (2008). Transcriptional control of human p53-regulated genes.. Nat Rev Mol Cell Biol.

[49] Espinosa JM (2008). Mechanisms of regulatory diversity within the p53 transcriptional network.. Oncogene.

[50] Perez CA, Ott J, Mays DJ, Pietenpol JA (2007). p63 consensus DNA-binding site: identification, analysis and application into a p63MH algorithm.. Oncogene.

[51] Osada M, Park HL, Nagakawa Y, Yamashita K, Fomenkov A (2005). Differential recognition of response elements determines target gene specificity for p53 and p63.. Mol Cell Biol.

[52] Jegga AG, Inga A, Menendez D, Aronow BJ, Resnick MA (2008). Functional evolution of the p53 regulatory network through its target response elements.. Proc Natl Acad Sci USA.

[53] Zhang X, Krutchinsky A, Fukuda A, Chen W, Yamamura S (2005). MED1/TRAP220 exists predominantly in a TRAP/Mediator subpopulation enriched in RNA polymerase II and is required for ER-mediated transcription.. Mol Cell.

[54] Olivier M, Langerod A, Carrieri P, Bergh J, Klaar S (2006). The clinical value of somatic TP53 gene mutations in 1,794 patients with breast cancer.. Clin Cancer Res.

[55] Brenton JD, Carey LA, Ahmed AA, Caldas C (2005). Molecular classification and molecular forecasting of breast cancer: ready for clinical application?. J Clin Oncol.

[56] Sorlie T, Tibshirani R, Parker J, Hastie T, Marron JS (2003). Repeated observation of breast tumor subtypes in independent gene expression data sets.. Proc Natl Acad Sci USA.

[57] Pal S, Datta K, Mukhopadhyay D (2001). Central role of p53 on regulation of vascular permeability factor/vascular endothelial growth factor (VPF/VEGF) expression in mammary carcinoma.. Cancer Res.

[58] Bian J, Sun Y (1997). Transcriptional activation by p53 of the human type IV collagenase (gelatinase A or matrix metalloproteinase 2) promoter.. Mol Cell Biol.

[59] Wang SE, Narasanna A, Whitell CW, Wu FY, Friedman DB (2007). Convergence of p53 and transforming growth factor beta (TGFbeta) signaling on activating expression of the tumor suppressor gene maspin in mammary epithelial cells.. J Biol Chem.

[60] Kunz C, Pebler S, Otte J, von der Ahe D (1995). Differential regulation of plasminogen activator and inhibitor gene transcription by the tumor suppressor p53.. Nucleic Acids Res.

[61] Teodoro JG, Parker AE, Zhu X, Green MR (2006). p53-mediated inhibition of angiogenesis through up-regulation of a collagen prolyl hydroxylase.. Science.

[62] Komarova EA, Krivokrysenko V, Wang K, Neznanov N, Chernov MV (2005). p53 is a suppressor of inflammatory response in mice.. Faseb J.

[63] Roger L, Gadea G, Roux P (2006). Control of cell migration: a tumour suppressor function for p53?. Biol Cell.

[64] Hyder SM, Nawaz Z, Chiappetta C, Stancel GM (2000). Identification of functional estrogen response elements in the gene coding for the potent angiogenic factor vascular endothelial growth factor.. Cancer Res.

[65] Ferrara N, Davis-Smyth T (1997). The biology of vascular endothelial growth factor.. Endocr Rev.

[66] Carmeliet P (2005). Angiogenesis in life, disease and medicine.. Nature.

[67] Chappel JC, Taylor SM, Ferrara N, Bautch VL (2009). Local Guidance of Emerging Vessel Sprouts Requires Soluble Flt-1.. Developmental Cell.

[68] Menendez D, Inga A, Resnick MA (2006). The biological impact of the human master regulator p53 can be altered by mutations that change the spectrum and expression of its target genes.. Mol Cell Biol.

