# Supplementary figures and images for: The Coordinated P53 and Estrogen Receptor Cis-Regulation at an FLT1 Promoter SNP Is Specific to Genotoxic Stress and Estrogenic Compound
Source: PLoS One. 2010 Apr 21;5(4):e10236. doi: 10.1371/journal.pone.0010236 (PMC2858160; doi:10.1371/journal.pone.0010236)

**Figure S1**

**A**


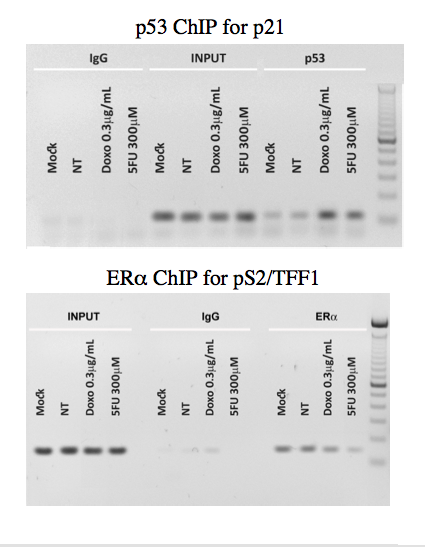


**B**


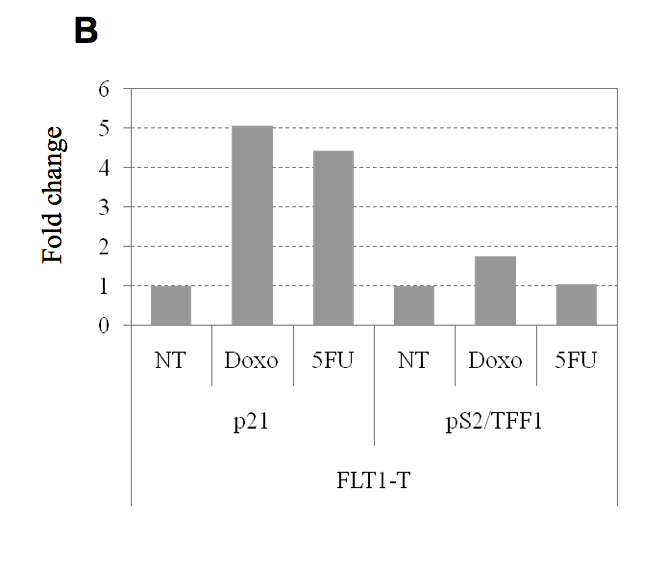

Supplement: Figure S1 — Impact of doxorubicin and 5FU on p53 and ER occupancy at target sites. (A): Doxorubicin and 5FU treatment result in a similar increase of p53 occupancy at the p21 promoter. (B) Doxorubicin and 5FU treatment showed a similar negative effect on ERα occupancy at the TFF1 promoter. Shown are representative PCR results obtained using template DNA retrieved from ChIP experiment conducted in MCF7 cells with the indicated primary antibodies and primers specific for the p53 RE and ERE1 containing regions of the target promoters. The effect of doxorubicin and 5FU were compared. In addition to a p53 specific Ab (DO1) and an ERα Ab (H-184 Santa Cruz) the IgG Ab was used as negative control. PCR of input DNA is also shown. (C) Fold change in site occupancy measured using real time PCR. Data are presented following the same order as in panel A. The antibody used for the ChIP experiment was targeted at p53 for the p21 promoter site and at ERα for pS2/TFF1 promoter site. (0.21 MB DOC) [file pone.0010236.s001.doc]

**Figure S2**


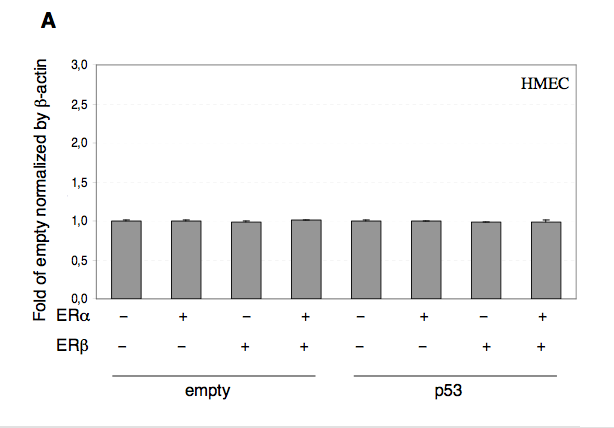


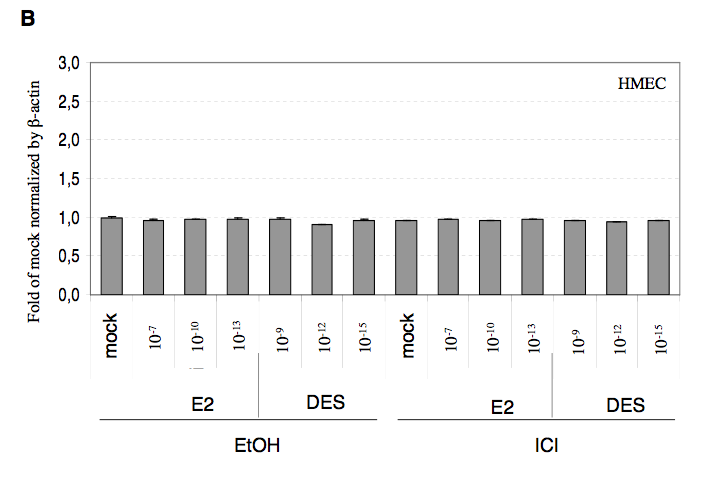

Supplement: Figure S2 — Quantification of FLT1 mRNA in response to p53, ER overexpression in HMEC cells. (A) Cells were transfected with expression vectors for p53, ERα or ERβ as indicated. (B) Cells co-transfected with p53, ERα and ERβ were also treated 24 hours after transfection by estrogen ligands (Estradiol, E2; Diethylstilbestrol, DES) at the indicated concentrations (M). When indicated, treatment included a 100-fold excess of the ER antagonist ICI 182,780. For both panels, histograms represent the average fold of induction relative to the beta-actin housekeeping gene, calculated using the ΔCt method. Error bars present the standard errors of at least three replicates. (0.09 MB DOC) [file pone.0010236.s002.doc]
