# Supplementary material for: The Coordinated P53 and Estrogen Receptor Cis-Regulation at an FLT1 Promoter SNP Is Specific to Genotoxic Stress and Estrogenic Compound
Source: PLoS One. 2010 Apr 21;5(4):e10236. doi: 10.1371/journal.pone.0010236 (PMC2858160; doi:10.1371/journal.pone.0010236)
Supplement: Table S1 — Primers for 2-round PCR mutagenesis of 1 Kb FLT1-T constructs and description of method. (0.05 MB DOC) [file pone.0010236.s003.doc]

**Table S1**

Table S1: primers for 2-round PCR mutagenesis of 1Kb FLT1-T constructs.

| 1kbFLT1-F | CAGCTGGGTACCTTACCCAACCTT |
| --- | --- |
| 1kbFLT1-R | AAGCTTCTCGAGTTCCCTGCGGCTACGGAGAG |
| ere2-F | ACAGTCTGAAGGAATCAGGAGCGGCGGGAC |
| ere2-R | GTCCCGCCGCTCCTGATTCCTTCAGACTGT |
| puERE1-F | GGTTGTGGTCAGTGTCCTTCATCTTGAGTA |
| puERE1-R | TACTCAAGATGAAGGACACTGACCACAACC |

*PCR-based site-specific mutagenesis*. A two-round PCR-based approach was used using primers presented in Suppl. Table 1. Each of the mutagenesis primer (*e.g.* ere1-F or ere1-R with complementary sequences) was used in combination with the appropriate external primer (*e.g.* 1kbFLT1-F for ere1-R), annealing either at the 5’ or 3’ end of the 1kb FLT1 fragment, in two PCR reactions that produced two amplicons overlapping by 30bp, corresponding to the sequence of mutagenesis primer carrying the desired mutation. The two PCR products were then used as template for a second PCR that utilized only the external pair of primers that contain non-homology tails with restriction sites (Xho I and Kpn I, underlined in Suppl. Table 1) for cloning. PCR products were purified (QIAquick kit, QIAGEN, Milan, Italy) digested by Xho I and Kpn I and ligated into an equally digested pGL3 promoter plasmid. Clones obtained upon transformation in *E. coli* cells were recovered and checked by restriction analysis and DNA sequencing.
