# Supplementary material for: The Coordinated P53 and Estrogen Receptor Cis-Regulation at an FLT1 Promoter SNP Is Specific to Genotoxic Stress and Estrogenic Compound
Source: PLoS One. 2010 Apr 21;5(4):e10236. doi: 10.1371/journal.pone.0010236 (PMC2858160; doi:10.1371/journal.pone.0010236)
Supplement: Table S2 — List of the primers (A), the probes (B) used in the Real Time PCR experiments and (C) the primers used in ChIP experiments. (0.05 MB DOC) [file pone.0010236.s004.doc]

**Table S2**: List of the primers (A), the probes (B) used in the Real Time PCR experiments and (C) the primers used in ChIP experiments.

A: primers for real-time PCR

| GAPDH-F | GAAGGTGAAGGTCGGAGTC |
| --- | --- |
| GAPDH-R | GAAGATGGTGATGGGATTTC |
| B2M-F | AGGCTATCCAGCGTACTCCA |
| B2M-R | ATGGATGAAACCCAGACACA |
| p21-F | CTGGAGACTCTCAGGGTCGAAA |
| p21-R | GATTAGGGCTTCCTCTTGGAGAA |
| FLT1-F | CCACCATCTGAACGTGGTTA |
| FLT1-R | GCTGCATCCTTGTTGAGAAA |

**B: 5’ FAM-labeled Taqman probes**

| GAPDH | 6FAM-CAAGCTTCCCGTTCTCAGCCXT--PH |
| --- | --- |
| B2M | 6FAM-TCACGTCATCCAGCAGAGAATGGA--TMR |
| p21 | 6FAM-CGGCAGACCAGCATGACAGATTTCTACCXT--PH |
| FLT1 | 6FAM-CTGGGAGCCTGCACCAAGCA--TMR |

C: primer for ChIP experiments

| p53-RE-T-F | TCCAAGTTGCAGGAGCAGTTT |
| --- | --- |
| p53-RE-T-R | AACTGACGCTTGCTTGGCA |
| FLT1-ERE1-F | CAGAGACAAGACTGGGCTGCT |
| FLT1-ERE1-R | CGTGAAACTGCTCCTGCAACT |
| FLT1-ERE2-F | CTCCAGGTTCAGTGCCTTGT |
| FLT1-ERE2-R | AAAGTCCGATCCAAGGGAAG |
